# A Specific Predicting Model for Screening Skip Metastasis From Patients With Negative Central Lymph Nodes Metastasis in Papillary Thyroid Cancer

**DOI:** 10.3389/fendo.2021.743900

**Published:** 2021-09-30

**Authors:** Zheyu Yang, Yu Heng, Qiwu Zhao, Zichao Cao, Lei Tao, Weihua Qiu, Wei Cai

**Affiliations:** ^1^ Department of General Surgery, Ruijin Hospital, Shanghai Jiao Tong University School of Medicine, Shanghai, China; ^2^ ENT Institute and Department of Otorhinolaryngology, Eye & ENT Hospital, Fudan University, Shanghai, China

**Keywords:** predicting, nomogram, N1b metastasis, papillary thyroid cancer, skip metastasis

## Abstract

Skip metastasis is a specific type of papillary thyroid cancer lymph node metastasis (LNM). The present study aimed to clarify the typical clinical characteristics of skip metastasis and optimize the prediction model, so as to provide a more individual treatment mode for skip metastasis. We retrospectively analyzed 1075 PTC patients with different lymph node metastasis statuses from two clinical centers. Comparisons have been made between patients with skip metastasis and other types of LNM. Univariate and multivariate analyses were performed to detect the risk factors for skip metastasis with negative LNM, and a nomogram for predicting skip metastasis was established. The rate of skip metastasis was 3.4% (37/1075). Compared with other types of LNM, significant differences showed in tumor size, upper portion location, thyroid capsular invasion, and ipsilateral nodular goiter with the central lymph node metastasis (CLNM) group, and in age and gender with the lateral lymph node metastasis (LLNM) group. Four variables were found to be significantly associated with skip metastasis and were used to construct the model: thyroid capsular invasion, multifocality, tumor size > 1 cm, and upper portion. The nomogram had good discrimination with a concordance index of 0.886 (95% confidence interval [CI], 0.823 to 0.948). In conclusion, the significant differences between skip metastasis and other types of LNM indicated that the lymph node drainage pathway of skip metastasis is different from either CLNM or LLNM. Furthermore, we established a nomogram for predicting risk of skip metastasis, which was able to effectively predict the potential risk of skip metastasis in patients without preoperative LNM clue.

## Introduction

Papillary thyroid cancer (PTC), accounting for 90% of thyroid cancer pathologies, is the most common type of cancer of the head and neck region, and its incidence is increasing worldwide (1–3). Although the mortality of PTC has not changed, and disease-specific mortality at 10 years is less than 5% (4, 5), the clinical manifestations of PTC patients are not always favorable (6). According to American Thyroid Association (ATA) in 2015, risk stratification, capsular invasion, extrathyroidal extension, lymph node metastasis (LNM), incomplete resection, distant metastasis, and vascular invasion are significant risk factors of tumor recurrence or progression (7, 8).

In different reports, PTC is shown to involve cervical lymph node metastasis in 20–50% of patients with macrometastasis and in up to 90% of patients with micrometastasis detected by sensitive detection methods (9–11). LNM of PTC occurs in a stepwise fashion. Spreading from the thyroid gland, the central and lateral lymph node compartments on the ipsilateral side of the thyroid tumor represent the first echelons of lymphatic drainage followed by the mediastinal and contralateral lateral lymph node compartments (12, 13). According to the American Joint Committee on Cancer (AJCC, 8th edition) staging system, LNM is divided into N1a (central lymph node metastasis, CLNM) and N1b (lateral lymph node metastasis, LLNM) (14).

Skip metastases, which leaps over the central compartment, is not uncommon in cases of PTC with lateral cervical lymph node metastasis. The frequency of skip metastasis has been reported to vary between 7.4% and 21.8% in PTC with lateral LNM (15–17). Skip metastases have also been reported in other tumors; the prognosis of skip metastases in different tumors is also significantly different (18–20). Previous studies have constructed several prediction models for the risk of skip metastasis, identifying patients with skip metastasis from that with LLNM, which had limited clinical value (15, 16, 21, 22). The purpose of the present study is to precisely describe the clinical characteristics of skip metastasis on the basis of previous studies and build a prediction model with more clinical significance.

## Methods

### Patient Cohort

Between June 2017 and June 2019, 1179 newly diagnosed primary PTC patients who underwent thyroidectomy at Department of General Surgery, Ruijin Hospital, Shanghai Jiao Tong University School of Medicine, and Department of Otorhinolaryngology, Head and Neck Surgery, at the Eye, Ear, Nose, and Throat (EENT) Hospital of Fudan University were enrolled in our study. All patients included in the study had pathologically confirmed PTC, and we excluded patients with poorly differentiated papillary cancer (PDTC). After exclusion of patients with no lymph nodes removed (n=33) or incomplete laboratory and pathological results (n=71), 1,075 patients who had pathological PTC and received thyroidectomy along with lymph node dissection were studied. 1075 patients included 381 males (35.4%) and 694 females (64.6%), with a mean age of 42.9 (18–71) years, and mean BMI of 23.81 (16.90-40.52) (**Table 1**). None of the patients had existing distant metastasis or other malignancies when diagnosed and all of them were confirmed as PTC by postoperative pathology.

**Table 1:** Demographics and clinical Characteristics of the cohort.

Variable	Value (%)
**Age**	
**mean**	42.9
**BMI**	
**mean**	23.73
**Gender**	
**male**	381 (35.4)
**female**	694 (64.6)
**Size of largest lesion (US)**	
**mean**	0.93
**medium**	0.9
**Thyroid capsule invasion (US)**	435 (40.5)
**Multifocality (US)**	332 (30.9)
**Bilateral disease**	236 (21.9)
**Negative LNM**	495 (46.1)
**mean harvested CLN**	5.1
**LNM**	580 (53.9)
**Skip metastasis**	37 (6.4)
** mean harvested CLN**	7.9
**CLNM**	383 (66)
** mean harvested CLN**	6.7
** mean positive CLN**	3.3
**LLNM**	160 (27.6)
** mean harvested CLN**	8.1
** mean positive CLN**	4.1

### Data Collection, Surgical Procedure, and Pathological Approach

Preoperative data sources including serum index, ultrasound examination (US) and fine-needle aspiration (FNA) were collected from Electronic Medical Records System for further analysis. Preoperative US and US-guide FNA were performed strictly according to Thyroid Imaging Reporting and Data System (TI-RADS), Preoperative US provided standardized description of the number of tumors, tumor location, tumor size, tumor edge distance from capsule(<0mm defined as capsular invasion), and lymph nodes involved. In addition to thyroid and parathyroid glands, a description of central and lateral lymph nodes was also included. All patients enrolled were identified as T0-4N0-1bM0 according to the 2015 American Joint Committee on Cancer (AJCC) Tumor Node Metastasis (TNM) staging system.

The basic surgical procedure consisted of a total lobectomy with a central compartment lymph node dissection (LND). The nodal tissue within the central compartment was removed from the level of the hyoid bone along the recurrent laryngeal nerves(RLN), and to a comparable level behind the clavicle. All resected tissues and lymph nodes were sent for intraoperative pathology examinations. For those with CLNM, bilateral thyroidectomy was performed simultaneously, but no elective lateral neck dissections were performed. Routine exploration of the ipsilateral neck was conducted for suspected level II, III, IV, and V lymph node metastasis.

All acquired specimens were examined by two or more board-certified pathologists from Shanghai Ruijin Hospital & Shanghai EENT Hospital. Pathological features analyzed were pathological type of tumor, type of the surrounding thyroid tissues, tumor size, capsular invasion, extrathyroidal extension, multifocality (more than one lesion in unilateral thyroid lobe), and lymph node metastasis.

### Statistical Analysis

Chi-square test and independent t-test were conducted for categorical variables and continuous variables respectively. Univariate and multivariate analyses were conducted for screening risk variables that were significantly associated with the skip metastases. P <0.05 was considered to indicate a statistically significant difference, and statistical analyses were conducted using the SPSS 24.0 package (SPSS Inc., Chicago, IL, USA). Variables with the p-value < 0.05 from the univariate logistic regression were then used for multivariate logistic regression to construct a risk prediction model – Nomogram, in R software (ver. 3.5.1, R Development Core Team). The discrimination and consensus degree of our newly-established predictive model were tested through the receiver operating characteristic (ROC) curve, the calibration curve, and the concordance index (C-index).

### Ethical Statement

This study was approved by the Institutional Ethics Committee of the Eye and ENT Hospital of Fudan University and Ruijin Hospital, Shanghai Jiao Tong University School of Medicine, and was also approved by Chinese Clinical Trial (ChiCTR2100043353). All participants gave informed consent to take part in the study after full explanation of the purpose and nature of all procedures used.

## Results

### Demographics and Clinicopathological Characteristics of Patients in the Cohort

The characteristics of the study patients are given in **Table 1**. Through preoperative US detection and fine needle aspiration biopsy (FNA), the mean tumor size was 0.93 cm with a range of 0.05-5.4 cm. Of the patients, 435 (40.5%) were diagnosed with thyroid capsule invasion, 332 (30.9%) were detected with multifocality, 236 (21.9%) were confirmed to have bilateral PTC, and 580 (53.9%) were ultimately confirmed to have LNM by postoperative pathology. In patients with LNM, 37 patients (6.4%) were diagnosed with skip metastasis, while 383 (66%) with CLNM and 160 (27.6%) with LLNM. The mean harvested central lymph nodes (CLN) in skip, CLNM, and LLNM were 7.9, 6.7, and 8.1, respectively, and the mean positive CLN in CLNM and LLNM were 3.3 and 4.1, respectively.

### The Comparison Between Patients With Skip Metastasis and Conventional Pathway of Metastasis (CLNM Only and CLNM Accompanied With Lateral Cervical Lymph Node Metastasis)

580 (54.0%) patients were confirmed to have positive cervical lymph node metastases in our cohort. We classified them into three subgroups according to the extent of neck metastases: Skip Group (patients with skip metastasis, n=37), CLNM Group (patients with CLNM only, n=383), and LLNM Group (patients with both CLNM and LLNM, n=160). Patients in Skip Group showed significantly larger tumor size than those in CLNM Group (P-value= 0.002), however, no such difference was found between Skip Group and LLNM Group (P-value> 0.05). More patients exhibited thyroid capsular invasion and ipsilateral nodular goiter in Skip Group than in CLNM Group (P-value= 0.004 and 0.022, respectively). As for tumor location, significant difference was found between Skip Group and CLNM Group: upper pole tumors were more common in Skip Group, and lower pole ones were relatively fewer. But no difference was shown between Skip Group and LLNM Group (P-value= 0.275). Moreover, a difference also existed between Skip Group and LLNM Group in age and gender (P-value= 0.009 and 0.018, respectively) (**Table 2**).

**Table 2:** The clinicopathological characteristics of PTC patients with LNM.

	CLNM Group		P value (Skip vs. CLNM)	Skip Group		P value (Skip vs. CLNM)	LLNM Group	
	n=383	%		n=37	%		n=160	%
**Age**			0.222			**0.009**		
>40	167	43.6		20	54.1		50	31.3
<=40	216	56.4		17	45.9		110	68.8
**Gender**			0.192			**0.018**		
Male	156	40.7		11	29.7		82	51.2
Female	227	59.3		26	70.3		78	48.8
**Maximum tumor diameter (mean ± SD)**	0.94 ± 0.62		**0.002**	1.29 ± 0.84		0.180	1.54 ± 1.09	
**Thyroid capsular invasion**			**0.004**			0.421		
No	178	46.5		8	21.6		45	28.1
Yes	205	53.5		29	78.4		115	71.9
**Bilateral disease**			0.397			0.454		
Absent	293	76.5		26	70.3		102	63.7
Present	90	23.5		11	29.7		58	36.3
**Multifocality**			0.884			0.190		
Absent	223	58.2		22	59.5		76	47.5
Present	160	41.8		15	40.5		84	52.5
**Tumor location**			**0.018**			0.275		
Isthmus	24	6.3		1	2.7		3	1.9
Upper portion	97	25.3		19	51.4		62	38.8
Middle portion	102	26.6		6	16.2		40	25.0
Lower portion	155	40.5		11	29.7		43	26.9
Diffuse TC	5	1.3		0	0.0		12	7.5
**PTC with ipsilateral Hashimoto thyroiditis**			0.182			0.570		
No	307	80.2		33	89.2		137	85.6
Yes	76	19.8		4	10.8		23	14.4
**PTC with ipsilateral nodular goiter**			**0.022**			0.486		
No	293	76.5		22	59.5		85	53.1
Yes	90	23.5		15	40.5		75	46.9

Bold: P < 0.05.

### Univariate and Multivariate Logistic Regression for Identifying Independent Risk Factors of Skip Metastasis in Patients With No Central Lymph Node Metastasis

Main clinicopathological characteristics of patients with no CLNM were shown in **Table 3**. Significantly more patients exhibited thyroid capsular invasion (P-value= 0.000) and bilateral disease (P-value= 0.025) in Skip Group than in Negative LNM Group, and multifocality was also more common in Skip Group (P-value= 0.000). Factors including age >40, gender, PTC with ipsilateral Hashimoto thyroiditis, and nodular goiter all showed no significant difference between these two subgroups (P-value> 0.05). In all patients with no central lymph node metastasis, the presence of thyroid capsular invasion, bilateral disease and multifocality, tumor in the upper portion, and maximum tumor diameter >=1cm are significantly associated with skip metastasis in the univariate analysis. Those elements were then enrolled into the multivariate logistic regression analysis. As a result, a total of four variables were confirmed as independent risk factors of skip metastasis in patients with no central lymph node metastasis: thyroid capsular invasion, multifocality, tumor in the upper portion, and maximum tumor diameter >=1cm (**Table 4**).

**Table 3:** The clinicopathological characteristics of PTC patients with no CLNM.

	Negative LNM Group		Skip Group		P value
	n=495	%	n=37	%	
**Age**					0.418
>40	301	60.8	20	54.1	
<=40	194	39.2	17	45.9	
**Gender**					0.685
Male	132	26.7	11	29.7	
Female	363	73.3	26	70.3	
**Maximum tumor diameter(**mean ± SD**)**	0.69 ± 0.55		1.29 ± 0.84		***0.000***
**Thyroid capsular invasion**					***0.000***
No	409	82.6	8	21.6	
Yes	86	17.4	29	78.4	
**Bilateral disease**					***0.025***
Absent	418	84.4	26	70.3	
Present	77	15.6	11	29.7	
**Multifocality**					***0.000***
Absent	422	85.3	22	59.5	
Present	73	14.7	15	40.5	
**Tumor location**					***0.021***
Isthmus	29	5.9	1	2.7	
Upper portion	136	27.5	19	51.4	
Middle portion	174	35.2	6	16.2	
Lower portion	148	29.9	11	29.7	
Diffuse PTC	8	1.6	0	0.0	
**PTC with ipsilateral Hashimoto thyroiditis**					0.131
No	390	78.8	33	89.2	
Yes	105	21.2	4	10.8	
**PTC with ipsilateral nodular goiter**					0.095
No	358	72.3	22	59.5	
Yes	137	27.7	15	40.5	

Bold: P < 0.05.

**Table 4:** Univariate and multivariate logistic analyses of skip LNM for PTC patients with no CLNM.

	Univariate analysis		Multivariate analysis	
	Hazard ratio (95% CI)	P value	Hazard ratio (95% CI)	P value
**Factors selected**				
**Age**		0.419		
>40 vs. <=40	0.758 (0.388-1.484)			
**G**ender		0.685		
Male vs. Female	1.163 (0.559-2.420)			
**BMI**		0.797		
>=23.0 vs. <23.0	0.916 (0.470-1.788)			
**Thyroid capsular invasion**		***0.000***		***0.000***
Yes vs. No	17.240 (7.619-39.007)		12.456 (5.304-29.247)	
**Location**		***0.003***		***0.013***
Upper portion vs. non-upper portion	2.786 (1.420-5.468)		2.736 (1.242-6.028)	
**Maximum tumor diameter**		***0.000***		***0.001***
>=1.0cm vs. <1.0cm	6.096 (3.048-12.191)		4.026 (1.820-8.908)	
**Bilateral disease**		***0.029***		0.332
Present vs. Absent	2.297 (1.090-4.841)		1.573 (0.629-3.934)	
**Multifocality**		***0.000***		***0.008***
Present vs. Absent	3.941 (1.954-7.951)		3.110 (1.345-7.187)	
**PTC with ipsilateral Hashimoto thyroiditis**		0.140		
Yes vs. No	0.450 (0.156-1.299)			
**PTC with ipsilateral nodular goiter**		0.099		
Yes vs. No	1.782 (0.898-3.535)			

Bold: P < 0.05.

## Nomogram for Predicting Probability of Skip Metastasis and Validation of the Nomogram

**Figure 1:**
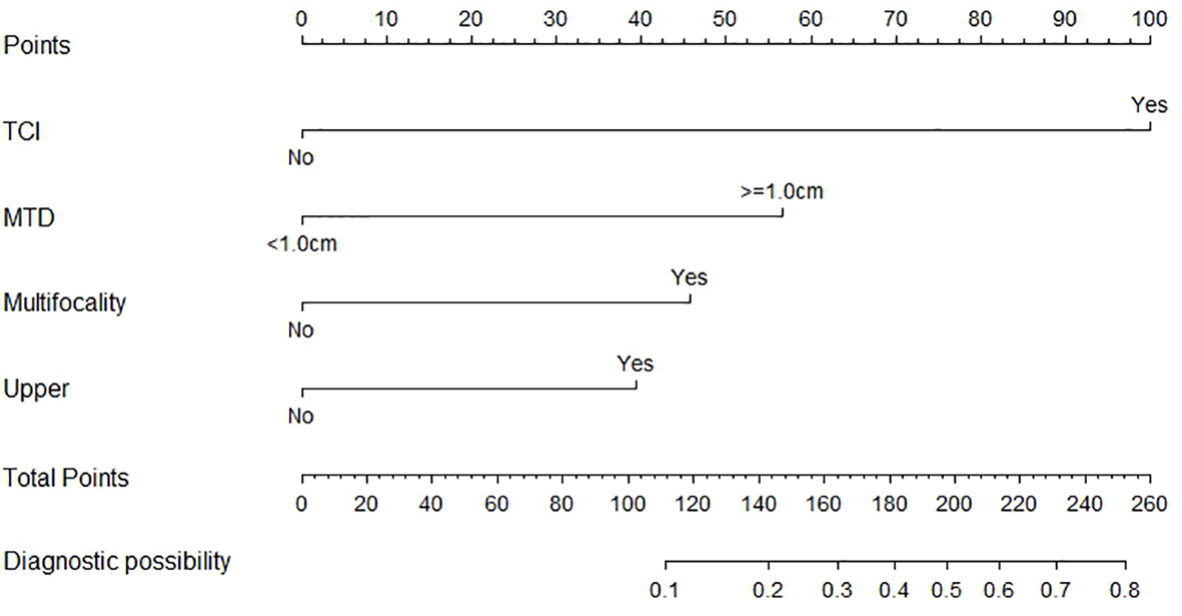
The nomogram for predicting risk of skip metastasis in PTC patients.

**Figure 2:**
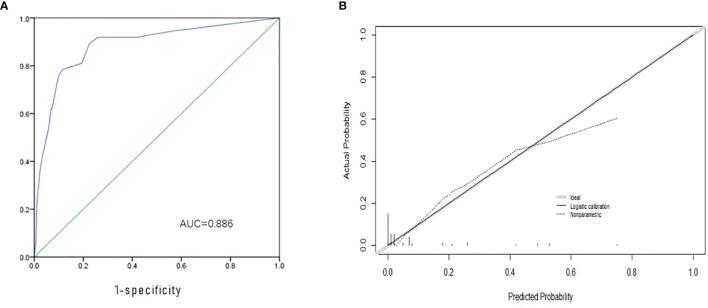
Evaluation and Validation of the nomogram. **(A)** The ROC curve and AUC of the nomogram; ROC, receiver operating characteristics; **(B)** The calibration curve of the nomogram for predicting possible CNM. Actual probability is plotted on the y-axis, and nomogram predicted probability on the x-axis.

Based on the independent factors screened through multivariate analysis, a nomogram was established for predicting individual risk of skip metastasis. The risk of each factor, including thyroid capsular invasion (TCI), maximum tumor diameters (MTD), multifocality, and upper portion, was quantified in our predicting model (shown in **Figure 1**) to predict the presence of skip metastasis from patients with negative LNM. For evaluating our nomogram’s ability to predict skip metastasis in PTC patients, we conducted an internal validation using 1,000 bootstrap resamples. As a result, C-index was found to be 0.886 (95% CI, 0.823 to 0.948), and 0.888 (95% CI, 0.827–0.948) after bootstrapping (**Figure 2A**). Furthermore, we also conducted a calibration plot for our newly-established model, and a favorable agreement was shown between the actual and estimated probability of skip metastasis (**Figure 2B**).

## Discussion

Skip metastasis to the lateral lymph nodes compartment, which leaps over the central compartment, was found in 7.4% to 19% of cases of PTC with LLNM, and researchers have focused on skip metastasis and studied the risk factors of its occurrence in several previous studies (13, 15–17). Although relevant studies proposed clinical features of skip metastasis and provided preliminary prediction models, they have common shortcomings in clinical significance and application potential. In the present study, we conducted comparative analysis of skip metastasis to describe its clinical characteristics more comprehensively. In addition to exploring the differences between patients with skip metastasis and other types of lymph node metastases to find the typical characteristics, we further constructed a specific prediction model focusing on screening present and potential skip metastasis from patients with negative CLNM, which might have more potentiality in clinical application.

We compared and analyzed group skip with other types of LNM in order to study the differences in clinical characteristics between group skip and group CLNM/LLNM. Significant differences in maximum tumor diameter, capsular invasion, tumor location, and ipsilateral nodular goiter between group skip and group CLNM, and in age and gender between group skip and group LLNM, were found. Compared with group CLNM, group skip has relatively similar clinical characteristics with group LLNM, however, this does not mean that skip metastasis and LLNM can be simply classified as the same type of LNM. In another multi-center clinical research with prognostic data, we found that the tumor recurrence survival rate of skip metastasis was significantly different from that of LLNM, which was demonstrated for the first time in PTC. Qiu et al. (23) indicated that skip metastasis might be related to less aggressive PTC and better prognosis in their systematic review and meta-analysis. Recognizing the above clinical characteristics is of great help to clarify the cause of skip metastasis and the lymph node metastasis pattern of PTC.

Moreover, we found that skip metastasis and LLNM both have obvious characteristics of upper pole tumor origin. Although there is no significant difference between skip metastasis and LLNM in terms of tumor location (**Table 3**), we found that patients with skip metastasis have a larger proportion of tumor located in the upper pole of the thyroid gland. Previous studies have reported this characteristic of skip metastasis (13, 22, 24); this is related to the special lymph node drainage pathway of the thyroid gland, which means that the first cite of lymph node drainage in a different area of the thyroid may be different. From this perspective, although PTC patients with skip metastasis and LLNM have the same characteristic of upper pole tumors, they have different clinical significance and prognostic characteristics. According to the 8th AJCC TNM staging system (14), patients with lateral lymph node metastasis (including skip metastasis) should be classified as N1b, however, whether skip metastasis should be simply classified as N1b remains to be further studied.

Separately from the previous studies, we performed univariate and multivariate analysis between group skip and group negative LNM. Predicting metastasis rather than skip, in skip metastasis, was the reason that the assessment model could make sense in clinical practice. Researchers tended to compare and analyze the skip group with the LLNM group (15, 16, 24, 25), while the predicting models did not have specific predictive values, because of the same surgical procedure and treatment strategy for skip metastasis or LLNM patients. However, identifying the present and potential skip metastasis patients from patients without preoperative lymph node metastasis features might provide more individual treatment strategies, including prophylactic lymph node exploration, Postoperative ^131^I treatment, and closer follow-up on lymph nodes. In this study, capsular invasion, primary tumor in the upper portion, maximum tumor diameter >1cm, and multifocality are independent risk factors for the occurrence of skip metastasis. The risk factors we obtained were different from the results in previous studies on skip metastasis, but closer to the predicting models for CLNM/LLNM (26–28). Therefore, we established a new nomogram to evaluate and quantify the risk of skip metastasis. In addition to confirming the patients with definite preoperative metastasis, more importantly, patients with high-risk characteristics of skip metastasis, who may have occult metastasis with negative LNM by preoperative ultrasound, were screened out.

In this research, we thoroughly studied the risk factors and clinical characteristics of skip metastasis. Compared with PTC patients with negative lymph node metastasis, patients with characteristics of capsular invasion, primary tumor in the upper portion, maximum tumor diameter >1cm, and multifocality are more likely to suffer skip metastasis. As a result, we could screen out the at-risk patients of skip metastasis from the negative LNM group, which played an important role in making an individual surgical option, reducing the incidence of secondary operations, formulating accurate active surveillance strategy, etc. At the same time, by comparing the similarities and differences between skip metastasis and other types of LNM, we found that tumor location played an important role in skip metastasis, which led to conjectures on the unique lymphatic metastasis pathway of skip metastasis. To sum up, we have made a comprehensive observation and analysis of skip metastasis, explaining its causes, drainage routes, risk factors, and possible prognosis.

However, there are limitations to our current study. Firstly, although the sample size of this retrospective study was not small, there were only 37 cases of skip metastasis, which might lead to bias in the process of statistical analysis. For the same reason, we could not perform external validation to further validate our prediction model. Secondly, we did not further study each risk factor, especially the location of the tumor, which will be explained in more detail in our other studies. Thirdly, the present study lacks long-term follow-up and study on the prognosis of skip metastasis, and there are few studies which could be referred to at present. Therefore, we will conduct in-depth studies on the prognosis of skip metastasis in future studies in order to clarify the mechanism and mode of skip metastasis more completely.

## Conclusion

There are significant differences between skip metastasis and other types of LNM in clinical characteristics, indicating that the lymph node drainage pathway of skip metastasis is different from either CLNM or LLNM. Furthermore, we established a nomogram for predicting the risk of skip metastasis, which was able to effectively predict the present and potential risk of skip metastasis in patients without preoperative LNM clue.

## Data Availability Statement

The raw data supporting the conclusions of this article will be made available by the authors, without undue reservation.

## Ethics Statement

Written informed consent was obtained from the individual(s) for the publication of any potentially identifiable images or data included in this article.

## Author Contributions

The article was written by ZY, YH, and QZ and they contributed equally to this work. WC, WQ, and LT provided guidance to the manuscript preparation. All authors contributed to the article and approved the submitted version.

## Funding

This research was supported by the Shanghai Municipal Science and Technology Commission (19441905400) and Shanghai Jiaotong University (YG2019ZDA15).

## Conflict of Interest

The authors declare that the research was conducted in the absence of any commercial or financial relationships that could be construed as a potential conflict of interest.

## Publisher’s Note

All claims expressed in this article are solely those of the authors and do not necessarily represent those of their affiliated organizations, or those of the publisher, the editors and the reviewers. Any product that may be evaluated in this article, or claim that may be made by its manufacturer, is not guaranteed or endorsed by the publisher.
